# Role of Magnetic Resonance Imaging in the Preoperative Staging and Work-Up of Patients Affected by Invasive Lobular Carcinoma or Invasive Ductolobular Carcinoma

**DOI:** 10.1155/2018/1569060

**Published:** 2018-06-26

**Authors:** Valeria Selvi, Jacopo Nori, Icro Meattini, Giulio Francolini, Noemi Morelli, Diego Di Benedetto, Giulia Bicchierai, Federica Di Naro, Maninderpal Kaur Gill, Lorenzo Orzalesi, Luis Sanchez, Tommaso Susini, Simonetta Bianchi, Lorenzo Livi, Vittorio Miele

**Affiliations:** ^1^Diagnostic Senology Unit, Azienda Ospedaliero-Universitaria Careggi, University of Florence, Florence, Italy; ^2^Radiation Oncology Unit, Azienda Ospedaliero-Universitaria Careggi, University of Florence, Florence, Italy; ^3^University of Malaya, Kuala Lumpur, Malaysia; ^4^Breast Surgery Unit, Azienda Ospedaliero-Universitaria Careggi, University of Florence, Florence, Italy; ^5^Department of Gynecology, Perinatology and Human Reproduction, University of Florence, Florence, Italy; ^6^Division of Pathological Anatomy, University of Florence, Florence, Italy; ^7^Department of Emergency Radiology, Azienda Ospedaliero-Universitaria Careggi, University of Florence, Italy

## Abstract

**Purpose:**

The prevalence of invasive lobular carcinoma (ILC), the second most common type of breast cancer, accounts for 5%–15% of all invasive breast cancer cases. Its histological feature to spread in rows of single cell layers explains why it often fails to form a palpable lesion and the lack of sensitivity of mammography and ultrasound (US) to detect it. It also has a higher incidence of multifocal, multicentric, and contralateral disease when compared to the other histological subtypes. The clinicopathologic features and outcomes of Invasive Ductolobular Carcinoma (IDLC) are very similar to the ILC. The purpose of our study is to assess the importance of MRI in the preoperative management and staging of patients affected by ILC or IDLC.

**Materials and Methods:**

We identified women diagnosed with ILC or IDLC. We selected the patients who had preoperative breast MRI. For each patient we identified the areas of multifocal, multicentric, or contralateral disease not visible to standard exams and detected by preoperative MRI. We analyzed the potential correlation between additional cancer areas and histological cancer markers.

**Results:**

Of the 155 women who met our inclusion criteria, 93 (60%) had additional cancer areas detected by MRI. In 61 women, 39,4% of the overall population, the additional cancer areas were confirmed by US/tomosynthesis second look and biopsy. Presurgical MRI staging changed surgical management in the 37,4% of the patients. Only six patients of the overall population needed a reoperation after the initial surgery. No statistically significant correlation was found between MRI overestimation and the presence of histological peritumoral vascular/linfatic invasion. No statistically significant correlation was found between additional cancer areas and histological cancer markers.

**Conclusions:**

Our study suggests that MRI is an important tool in the preoperative management and staging of patients affected by lobular or ductolobular invasive carcinoma.

## 1. Introduction

Breast cancer is the most frequent cancer in USA, with an estimated incidence of 296.980 new cases in 2013. The lifetime risk of developing a breast cancer is about 12%; yearly screening mammograms are proposed in asymptomatic women with age > 40 [1, 2].

Invasive lobular carcinoma (ILC) is the second most common type of breast cancer. Its prevalence accounts for 5–15% of all invasive breast cancers, with a maximum incidence in postmenopausal women. It has been found that the mean age of incidence is three years older than that of women affected by invasive ductal carcinoma (IDC) [3].

ILC has a typical histological growth behaviour. It arises from lobular epithelium and spreads as a single row of malignant cells along the breast ducts (Indian file manner), with weak desmoplastic reaction in surrounding connective stroma [3].

Due to these histological features, ILC often fails to present as a clinically palpable lesion, and it is often seen to spread diffusely through the breast stroma on mammography. Moreover, ILC spreading diffusely through the breast stroma leads to lower tendency to form round and circumscribed masses, only seen in 1%–3% of cases of ILC. Thus, ultrasound is more sensitive in detecting ILC [4, 5], with a reported sensitivity ranging from 68 to 98% [6].

On mammography, ILC is commonly characterized by the presence of asymmetry and architectural distortions with absence of calcifications [3]. Moreover ILC also tends to be isodense to normal adjacent breast parenchyma [5].

In view of these factors, mammography and ultrasound resulted in decreased diagnostic accuracy for ILC, with reported sensitivity ranging between 57 and 81% [4, 5].

MRI has a high sensitivity in the detection of breast cancer (over 90%) and it is well known for its increased diagnostic value in detecting multifocal, multicentric, or contralateral disease unrecognized on conventional exams [4]. Schelfout et al. reported that MRI detected 96% of multifocal/multicentric disease, while mammography and ultrasound only detected 28.6% and 26.5% respectively [7].

However, MRI also has a low specificity in detecting breast cancer [4], which can result in overtreatment (i.e., extensive surgery procedures), with no added advantages in terms of clinical outcome [8]. Furthermore, due to its limited availability and high cost [9], MRI is therefore best reserved only to a selected subgroup of patients.

MRI is a suitable diagnostic examination in the preoperative work-up and staging of ILC patients, due to the higher incidence of multifocal, multicentric, and contralateral disease, if compared to other histological subtypes [4]. However, the existing literature about this topic is rather sparse [10]. Ductolobular invasive carcinoma (IDLC) has similar clinic-pathologic features to ILC, with comparable outcomes [11].

For this reason, both ILC and IDLC were included in this analysis.

The aim of the current study is to assess the role of MRI in the preoperative staging and work-up of patients affected by ILC or IDLC.

## 2. Materials and Methods

The current study was a retrospective review of 163 patients. We included in the study all the patients with breast ILC or IDLC who had MRI studies prior to undergoing surgical therapy between January 2010 and July 2015, at the Breast Unit of Careggi Hospital, in Florence. Exclusions criteria were preoperative chemotherapy/radiotherapy administration or missing data.

Results of mammography (MRX), ultrasound (US), and MRI examinations of each patient were retrospectively reviewed, identifying areas of multifocal, multicentric, or contralateral disease detected only with MRI and not with standard exams (MRX or US). The results of MRX, US, and MRI examinations were scored according to the Breast Imaging Reporting and Data System (BIRADS) [12].

Patients underwent bilateral MRX and US before the MRI; size and position of the index lesion on both examinations were recorded.

Mammographic images were obtained in two standard planes: mediolateral oblique and craniocaudal using a dedicated equipment (Mammomat 2000, Siemens, Erlangen, Germany; Mammomat 3000 Nova, Siemens, Erlangen, Germany; Selenia Dimensions Hologic Inc., Bedford, USA).

Sonographic examination was performed using a broadband 10–13 Mhz linear transducer (Technos Mylab 70 XS; Esaote; Genoa, Italy).

All the MRI examinations were performed in prone position, with dedicated breast coils; A 1.5-Tesla equipment was used (Symphony®, Siemens Medical System, Erlangen, Germany; Philips Medical Systems, DA Best, The Nederland; Magnetom Avanto®, Siemens Medical System, Erlangen, Germany).

The size and position of the index lesion as well as any additional cancer areas detected on MRI were recorded. Regarding the size, the average diameter was chosen as the sizing reference for each lesion. The rate of change in the surgical management in view of the preoperative MRI findings was also recorded. Furthermore, reexcision rate after surgery was evaluated.

Histological diagnosis on surgical specimen performed at the local pathology department was reviewed; the data on the size of the index lesion and its histopathological features were assessed. The presence of peritumoral vascular/lymphatic invasion, ER, PGR and C-erb-2 status, and Ki67 were also collected.

## 3. Statistical Analysis

*t*-test was used to evaluate the significance of the differences observed using different diagnostic methods.

## 4. Results

Eight out of 163 women were excluded from the analysis (3 due to preoperative chemotherapy administration, 5 because of missing data). Thus the population of our study was composed of 155 patients.

Baseline characteristics of the population are summarized in Table 1.

**Figure 1:**
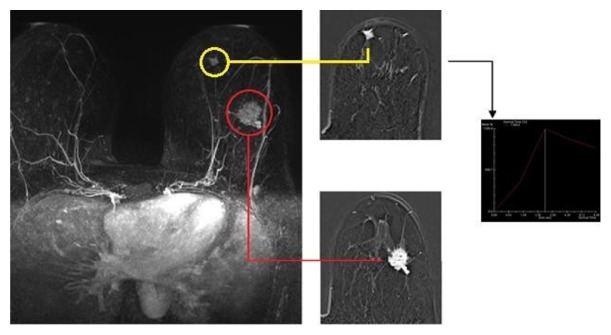
Index lesion (red circle; left SEQ, 28 mm), already documented with mammography and ultrasound. MRI leads to the detection of multicentric disease, confirmed to US second look and biopsy (yellow circle: left IIQ, 9 mm) (SEQ: Superior External Quadrant; IIQ: Inferior Internal Quadrant).

**Figure 2:**
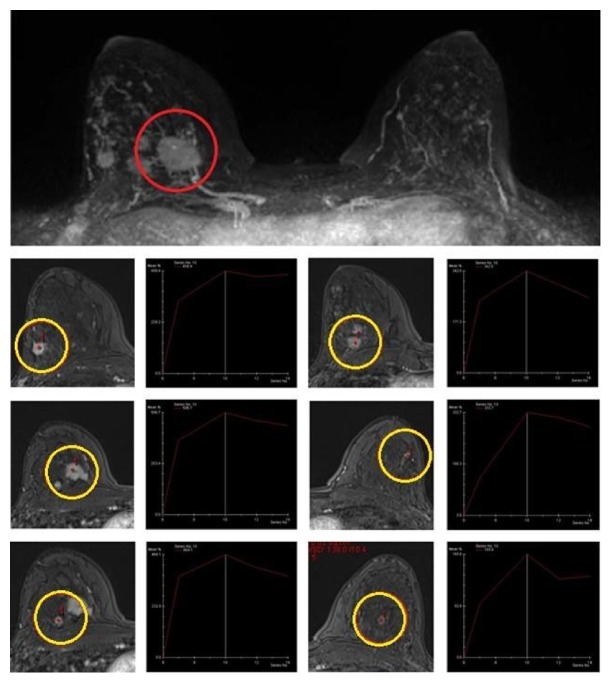
Index lesion (red circle; right SIQ, 27 mm), already documented with ultrasound. MRI leads to the detection of multifocal and multicentric disease, confirmed to US second look and biopsy (yellow circles: right SIQ, 6mm; right SEQ, 11mm; right CEQ, 10 mm; right CEQ dx, 11 mm; right CEQ dx, 5mm). Subcentimetric mass enhancement in the left breast resulted as negative to US second look. SEQ: Superior External Quadrant; SIQ: Superior Internal Quadrant; CEQ: Central External Quadrant.

**Figure 3:**
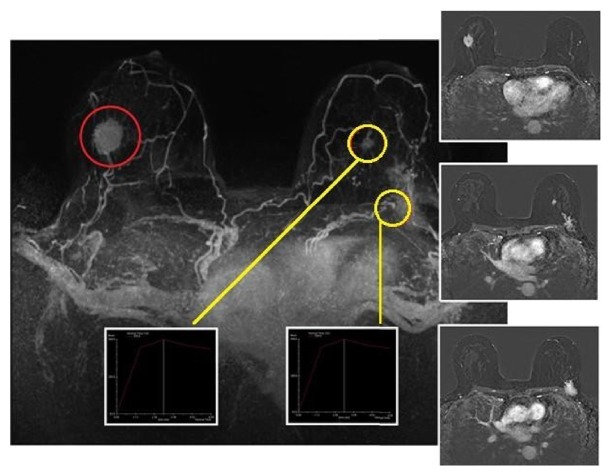
Index lesion (red circle; right IEQ, 30 mm), already documented with mammography and ultrasound. MRI leads to the detection of contralateral disease, confirmed to US second look and biopsy (yellow circle: left CEQ, 40 mm; left CEQ, 13 mm) (Inferior External Quadrant; IEQ: CEQ: Central External Quadrant).

When compared to MRX and US, MRI detected additional cancer areas in 93 out of 155 patients (60% of the overall population). Of these, additional cancer areas were confirmed with both US/tomosynthesis on second look and biopsy in only 61 patients (39,4% of the overall population; multifocal/multicentric and contralateral disease were found in 29,7% and 9,7% of patients, respectively). Presurgical MRI staging changed surgical management in the 37,4% of the patients; 27,4% underwent a wider exeresis/mastectomy instead of initially planned breast-conservative surgery, and 9,7% required also contralateral surgery. Only six patients of the overall population needed a reoperation after the initial surgery: mastectomy was performed in 5 patients because of positive margins after breast-conservative surgery, while one patient required bilateral mastectomy after breast-conservative surgery, due to the presence of BRCA1 mutation. Among the patients who needed to be reoperated on because of positive margins, three patients have had diagnosis of additional cancer area with the MRI performed before the initial surgery, later confirmed with US/tomosynthesis on second look and biopsy. Instead the other two presented the index lesion only and MRI had not added any further diagnostic information to MRX and US. Regarding the false positive patients, in whom the additional cancer areas detected by MRI were not confirmed on US/tomosynthesis second look, none of them presented a local recurrence. MRI performances are summarized in Table 2; in the Appendix there is the MRI documentation of three of the patients studied (Figures 1–3).

Average size of index lesion was 18 mm (range 2–40 mm), 14 mm (range 4–60 mm), and 22 mm (range 6–85 mm) on preoperative MRX, US, and MRI, respectively

The average size of the index lesion measured on surgical specimen was 17 mm (range 2,3–75mm). Difference of lesion size was significantly lower for mammography when compared to US and MRI; US showed a size underestimation rate of 18% while the MRI demonstrated an overestimation rate of 26% and mammography an overestimation rate of only 5% (p<0,001) (Table 3).

Overall sensitivity and specificity of MRI in this setting were 91,04% and 92,4%, respectively. No correlation was found between MRI overestimation and the presence of histological peritumoral vascular/linfatic invasion. No correlation was found in the presence of additional areas detected by MRI and ER status (p=0,103), PGR status (p=0,218), Ki67 (0,668), or C-erb-2 status (p=0,955) (Table 4).

## 5. Discussion

Results from the current analysis showed that if compared to MRX and US, preoperative MRI detected additional disease in 39,4% of patients, with 29,7% and 9,7% of the patients showing ipsilateral or contralateral undetected areas, respectively. Preoperative MRI had an overall sensitivity of 91,04%, confirming data from literature demonstrating the good performance of this examination in the preoperative setting of ILC and IDLC. Indeed previous series reported a sensitivity of 95% for MRI. [9, 13, 14].

These results therefore support the previous literature data on the superiority of MRI in detecting multifocal, multicentric, and contralateral disease, when compared to MRX and US [9, 15].

Due to the typical growth pattern of ILC/IDLC, with increased likelihood of multifocal, multicentric, and contralateral disease [10], MRI could have a key role in preoperative staging of these patients. In the 37,4% of our patients a change in surgical management was documented. Thus, targeted use of MRI in patients with ILC/IDLC could improve surgical planning, leading a lower rate of reoperation. Other authors achieved similar results too. A population based study conducted on the SEER database showed that preoperative MRI in this setting yielded a better surgical planning [8]. A meta-analysis of 18 studies reported that MRI detected additional disease in 32% of patients, with a subsequent change in surgical management in 28% of women [13]. More recent retrospective reports on ILC patients confirmed that high sensitivity of preoperative MRI in detecting multicentric and contralateral disease yielded a more appropriate surgical management plan [10].

Summarizing, the main aim of our analysis was testing the hypothesis that certain ILC/IDLC histological features could lead to a particular growth pattern, in which MRI could have an increased diagnostic sensitivity. The current study confirms our starting hypothesis.

Data from previous reports have confirmed the advantages of MRI in the preoperative assessment of patients with ILC [5, 6, 8, 10, 11, 15–20].

Furthermore, not all the authors agree that MRI improved sensitivity translated into short-term surgical outcome or long-term patient benefit [21].

This has also been reflected in the disagreements seen between the different guidelines in recent years; European Society of Breast Imaging [22] and EUSOMA working group [23] guidelines suggest a strong recommendation for the use of preoperative MRI for ILC. However, the American College of Radiology guidelines [24], later updated in 2013 [25], do not provide any recommendation about the use of preoperative MRI in patients affected by ILC, reporting insufficient evidence about this topic.

We evaluated also the size of the lesions and the difference between diagnostic imaging.

In the current study, the size of the lesion is significantly overestimated and underestimated with MRI and US, respectively, when compared to average size measured on surgical specimen; instead lesion size measured on MRX and the surgical specimen were relatively similar.

In the previous literature reviews, other authors have emphasised the trend for MRI overestimation of lesion size [3, 11, 26]. Conversely, some reports suggested that MRI could have a higher accuracy in determining tumour size if compared to MRX and US [7, 27].

We hypothesized the MRI overestimation we detected,could be explained by the presence of histological peritumoral vascular/lymphatic invasion. However, no correlation was found between MRI overestimation and this histological feature. To our knowledge, this is the first study testing the correlation between MRI overestimation of the size and histological features of the index lesion. We hypothesized also that the presence of additional cancer areas detected by MRI could be correlated to the presence of certain histological cancer markers. To our knowledge, this is the first study testing this topic; anyway no correlation was documented.

## 6. Conclusion

Results from this study show that MRI is a useful tool in the preoperative staging and surgical planning of patients affected by ILC/IDLC. MRI is very sensitive in the detection of multifocal, multicentric, and contralateral disease; it provides additional diagnostic information that is missed with the standard imaging modalities (MRX, US). Thus the targeted use of preoperative breast MRI in patients with a proven biopsy diagnosis of ILC or IDLC could significantly improve the surgical approach, allowing a more appropriate oncologic resection.

The retrospective nature of this study could anyway weaken these results. Prospective data on a larger study population are needed to better evaluate MRI performance in this setting; a randomized controlled trial is aimed to be organized in order to confirm the results that our study suggest.

## Figures and Tables

**Table 1:** Population description.

Number of patients	155

Median age (range)	53 (31–82)

Histology	ILC: 55%
	IDLC: 45%

Site of index lesion	SEQ: 49,3%
	SIQ: 14,6%
	IEQ: 13,2%
	IIQ: 6,8%
	CQ: 16,1%

ILC: Invasive Lobular Carcinoma; IDLC: Invasive Ductolobular Carcinoma; SEQ: Superior External Quadrant; SIQ: Superior Internal Quadrant; IEQ: Inferior External Quadrant; IIQ: Inferior Internal Quadrant; CQ: Central Quadrant; MRI: Magnetic Resonance Imaging.

**Table 2:** Summary of MRI performances.

	**Patients (%)**
Additional areas of disease found on MRI (%)	93 (60%)
Multifocal/multicentric disease (%)	46 (29,7%)
Additional cancer areas confirmed on second look exams and biopsy	61 (39,4%)
Contralateral disease	15 (9,6%)
Change in surgical management	58 (37,4%)
Reoperation rate	6 (3,9%)

**Table 3:** Comparison in lesion size.

	Average size (mm)	Range (mm)	Comparison with surgical specimen	p
MRX	18	2-40	+5%	
US	14	4-60	-18%	<0,001
MRI	21,25	6-70	+26%	

Surgical specimen	16,95	2,3-75	0	

**Table 4:** Relationship between additional cancer areas on MRI and Tumor histopathologic features.

**Additional cancer areas**	**Presence of additional cancer ** **areas**	**Absence of additional ** **cancer areas**	P
ER+ %	n of patients %	n of patients %	

≥ 80	98,1	98,7	
<80	1,9	1,3	
TOTAL	100	100	0,103

PgR+ %	n of patients %	n of patients %	

≥ 80	71,2	72,8	
<80	28,8	27,2	
TOTAL	100	100	0,218

HER2	n of patients %	n of patients %	

Positive 3+	3,6	6,3	
Negative 0/1+	52,7	53,2	
Doubt 2+	43,6	40,5	
Total	100	100	0,668
