# Corrigendum to “Role of Magnetic Resonance Imaging in the Preoperative Staging and Work-Up of Patients Affected by Invasive Lobular Carcinoma or Invasive Ductolobular Carcinoma”

**DOI:** 10.1155/2018/9056239

**Published:** 2018-09-06

**Authors:** Valeria Selvi, Jacopo Nori, Icro Meattini, Giulio Francolini, Noemi Morelli, Diego De Benedetto, Giulia Bicchierai, Federica Di Naro, Maninderpal Kaur Gill, Lorenzo Orzalesi, Luis Sanchez, Tommaso Susini, Simonetta Bianchi, Lorenzo Livi, Vittorio Miele

**Affiliations:** ^1^Diagnostic Senology Unit, Azienda Ospedaliero-Universitaria Careggi, University of Florence, Florence, Italy; ^2^Radiation Oncology Unit, Azienda Ospedaliero-Universitaria Careggi, University of Florence, Florence, Italy; ^3^University of Malaya, Kuala Lumpur, Malaysia; ^4^Breast Surgery Unit, Azienda Ospedaliero-Universitaria Careggi, University of Florence, Florence, Italy; ^5^Department of Gynecology, Perinatology and Human Reproduction, University of Florence, Florence, Italy; ^6^Division of Pathological Anatomy, University of Florence, Florence, Italy; ^7^Department of Emergency Radiology, Azienda Ospedaliero-Universitaria Careggi, University of Florence, Italy

In the article titled “Role of Magnetic Resonance Imaging in the Preoperative Staging and Work-Up of Patients Affected by Invasive Lobular Carcinoma or Invasive Ductolobular Carcinoma” [1], the name of the sixth author was given incorrectly as Diego Di Benedetto. The author's name should have been written as Diego De Benedetto. The revised authors' list is shown above.
